# Quality of life endpoints in cancer cachexia clinical trials: Systematic review 3 of the cachexia endpoints series

**DOI:** 10.1002/jcsm.13453

**Published:** 2024-03-29

**Authors:** Marianne J. Hjermstad, Gunnhild Jakobsen, Jann Arends, Trude R. Balstad, Leo R. Brown, Asta Bye, Andrew J.S. Coats, Olav F. Dajani, Ross D. Dolan, Marie T. Fallon, Christine Greil, Alexandra Grzyb, Stein Kaasa, Lisa H. Koteng, Anne M. May, James McDonald, Inger Ottestad, Iain Philips, Eric J. Roeland, Judith Sayers, Melanie R. Simpson, Richard J.E. Skipworth, Tora S. Solheim, Mariana S. Sousa, Ola M. Vagnildhaug, Barry J.A. Laird, the Cancer Cachexia Endpoints Working Group

**Affiliations:** ^1^ Department of Oncology Oslo University Hospital Oslo Norway; ^2^ European Palliative Care Research Centre (PRC), Department of Oncology, Oslo University Hospital, and Institute of Clinical Medicine University of Oslo Oslo Norway; ^3^ Department of Public Health and Nursing, Faculty of Medicine and Health Sciences Norwegian University of Science and Technology (NTNU) Oslo Norway; ^4^ Cancer Clinic, St. Olavs Hospital Trondheim University Hospital Trondheim Norway; ^5^ Department of Medicine I, Faculty of Medicine University of Freiburg Freiburg Germany; ^6^ Department of Clinical and Molecular Medicine, Faculty of Medicine and Health Sciences NTNU–Norwegian University of Science and Technology Trondheim Norway; ^7^ Department of Clinical Medicine, Clinical Nutrition Research Group, UiT The Arctic University of Norway Tromsø Norway; ^8^ Department of Clinical Surgery University of Edinburgh Edinburgh UK; ^9^ Royal Infirmary of Edinburgh Edinburgh UK; ^10^ Department of Nursing and Health Promotion, Faculty of Health Sciences OsloMet – Oslo Metropolitan University Oslo Norway; ^11^ Faculty of Medicine University of Warwick Coventry UK; ^12^ Academic Unit of Surgery University of Glasgow, Glasgow Royal Infirmary Glasgow UK; ^13^ Edinburgh Cancer Research Centre University of Edinburgh Edinburgh UK; ^14^ St Columba's Hospice Edinburgh UK; ^15^ Julius Center for Health Sciences and Primary Care, University Medical Center Utrecht Utrecht University Utrecht The Netherlands; ^16^ Department of Nutrition, Institute of Basic Medical Sciences University of Oslo Oslo Norway; ^17^ Department of Clinical Service, Division of Cancer Medicine, Section of Clinical Nutrition Oslo University Hospital Oslo Norway; ^18^ Oregon Health and Science University Knight Cancer Institute Portland OR USA; ^19^ Department of Public Health and Nursing Norwegian University of Science and Technology Trondheim Norway; ^20^ Department of Public Health and Nursing, Cancer Clinic, St Olavs Hospital Trondheim University Hospital Trondheim Norway; ^21^ Department of Clinical and Molecular Medicine Norwegian University of Science and Technology Trondheim Norway; ^22^ Improving Palliative, Aged and Chronic Care through Clinical Research and Translation (IMPACCT) University of Technology Sydney NSW Australia

**Keywords:** Cachexia, Cancer, Patient‐reported outcomes, Quality of life

## Abstract

The use of patient‐reported outcomes (PROMs) of quality of life (QOL) is common in cachexia trials. Patients' self‐report on health, functioning, wellbeing, and perceptions of care, represent important measures of efficacy. This review describes the frequency, variety, and reporting of QOL endpoints used in cancer cachexia clinical trials. Electronic literature searches were performed in Medline, Embase, and Cochrane (1990–2023). Seven thousand four hundred thirty‐five papers were retained for evaluation. Eligibility criteria included QOL as a study endpoint using validated measures, controlled design, adults (>18 years), ≥40 participants randomized, and intervention exceeding 2 weeks. The Covidence software was used for review procedures and data extractions. Four independent authors screened all records for consensus. Papers were screened by titles and abstracts, prior to full‐text reading. PRISMA guidance for systematic reviews was followed. The protocol was prospectively registered via PROSPERO (CRD42022276710). Fifty papers focused on QOL. Twenty‐four (48%) were double‐blind randomized controlled trials. Sample sizes varied considerably (*n* = 42 to 469). Thirty‐nine trials (78%) included multiple cancer types. Twenty‐seven trials (54%) featured multimodal interventions with various drugs and dietary supplements, 11 (22%) used nutritional interventions alone and 12 (24%) used a single pharmacological intervention only. The median duration of the interventions was 12 weeks (4–96). The most frequent QOL measure was the EORTC QLQ‐C30 (60%), followed by different FACIT questionnaires (34%). QOL was a primary, secondary, or exploratory endpoint in 15, 31 and 4 trials respectively, being the single primary in six. Statistically significant results on one or more QOL items favouring the intervention group were found in 18 trials. Eleven of these used a complete multidimensional measure. Adjustments for multiple testing when using multicomponent QOL measures were not reported. Nine trials (18%) defined a statistically or clinically significant difference for QOL, five with QOL as a primary outcome, and four with QOL as a secondary outcome. Correlation statistics with other study outcomes were rarely performed. PROMs including QOL are important endpoints in cachexia trials. We recommend using well‐validated QOL measures, including cachexia‐specific items such as weight history, appetite loss, and nutritional intake. Appropriate statistical methods with definitions of clinical significance, adjustment for multiple testing and few co‐primary endpoints are encouraged, as is an understanding of how interventions may relate to changes in QOL endpoints. A strategic and scientific‐based approach to PROM research in cachexia trials is warranted, to improve the research base in this field and avoid the use of QOL as supplementary measures.

## Introduction

Cancer cachexia is a multifactorial syndrome defined by an ongoing loss of skeletal muscle mass (with or without loss of fat mass), that cannot be fully reversed by conventional nutritional support and leads to progressive functional impairment.^1^ Cachexia in patients with cancer is very common,^2^ with a complex pathophysiology and multifaceted impact on patients. To date, there are no universally accepted endpoints for interventional cancer cachexia trials, and endpoints used remain highly variable. Yet if cancer cachexia is optimally treated, then this may have a direct or indirect effect on patients' quality of life (QOL) as studies have shown that improved nutritional status and/or an attenuation of inflammation correspond to improved QOL and well‐being, and better mental status.^3^, ^4^

The terms QOL and health related quality of life (HRQOL) are often used interchangeably^5^ as both denote the overall well‐being and health aspects in life. These cover broad topics, such as health status, physical functioning, symptoms, psychosocial adjustment, wellbeing, life satisfaction, and happiness,^6^ although some claim that HRQOL measures may more appropriately capture changes pertaining to health problems Broadly, both are multidimensional concepts representing an individual's perception of physical, psychological, and social aspects, and overall health (henceforth referred to as ‘QOL’). These QOL measures fall under the umbrella of patient‐reported outcome measures (PROMs) and regulatory agencies (US Food and Drug Administration (FDA),^7^ European Medicines Agency (EMA)^8^) recognizing PROMs as approvable endpoints in evaluating treatment efficacy^9^ in other conditions. To date, guidance on specific QOL measures as approvable endpoints in cachexia from regulatory agencies is not clear.

PROMs supplement clinician observations and objective findings with information based on patients' own lived experience. As such, the routine integration of PROMs within clinical research aligns with patient‐centred care, defined as ‘*care that is respectful of, and responsive to, individual patient preferences, needs and values, and ensuring that patient values guide all clinical decisions*’.^10^ PROMs have been utilized throughout cancer clinical trials, as endpoints, interventions, and prognostic markers. For example, PROMs defined the impact of integrating palliative care early in patients with advanced cancer demonstrating improved QOL, psychological distress, symptom burden,^11^ and a survival benefit.^12^ Additionally, empirical evidence indicates that PROMs provide independent prognostic information on survival in several cancer populations.^13^ Thus, using PROMs within clinical trials in patients with cancer is highly clinically relevant, is well accepted^9^ and particularly relevant to patients experiencing the multifaceted impacts of cancer cachexia.

Several types of PROMs exist, for example, the Edmonton Symptom Assessment System (ESAS),^14^ the M.D. Anderson Symptom Inventory (MDASI),^15^ the Spitzer Uniscale^16^ and the early Priestman and Baum LASA scales^17^ that all include assessments of wellbeing. Most QOL measures are multidimensional questionnaires, comprising several items that form specific scales, for example, physical, and emotional functioning, supplemented with single items. The questionnaires may measure generic QOL such as the Short Form‐36,^18^ and the EuroQol‐5D (EQ 5D)^19^ or may be disease‐ or condition‐specific, with the most frequent cancer‐specific PROMs being the Functional Assessment of Cancer Therapy scale (FACT‐G),^20^ the European Organization for Research and Treatment Quality of Life Questionnaire (EORTC QLQ‐C30),^21^ and the palliative care EORTC QLQ C15‐PAL,^22^ the early Rotterdam Symptom Checklist^23^ and the Japanese QOL‐ACD.^24^

In terms of what has been used to measure QOL in cachexia trials there are various assessments. The EORTC QLQ‐C30 is often supplemented with condition specific measures such as the one for Head‐and‐Neck Cancer^25^ corresponding to the FACIT condition specific measures^26^ used together with FACT‐G^20^ such as the FACT Fatigue and Anemia scales^27^ and the FACT Head and Neck Symptom inventory (FHNSI‐2).^28^ The content covered in these validated measures is relevant to patients with cachexia and they are commonly used together with more cachexia specific measures such as the first and subsequently revised Functional Assessment of Anorexia/Cachexia Therapy (FAACT) questionnaire^29^ and the EORTC QOL cancer cachexia questionnaire (EORTC QLQ‐CAX24).^30^ Despite these cachexia specific QOL assessments being available, there is no consensus about the most appropriate QOL endpoint in cachexia trials with inconsistency of assessments being used, analysis measures differing and subsequently varying reporting approaches. There is also no robust evidence to support which might be easiest to use in a trial and/or preferred by trial participants. These limitations are further compounded by the lack of a widely accepted ‘minimally clinically important difference (MCID)’, and this then impedes trial design and ultimately drug development.

This systematic review is part of a series of reviews assessing endpoints in cachexia clinical trials and aims specifically to examine QOL. The main objective was to describe the frequency and variety of QOL endpoints. This review includes descriptions of trial characteristics, interventions, QOL measures, reporting of QOL, and the relationship with significant primary and/or secondary outcomes.

## Methods

### Protocol and registration

This systematic review follows the Preferred Reporting Items for Systematic Reviews and Meta‐Analyzes Statement PRISMA (Supporting file S1).^31^

### Search strategy

The search for trials published from January 1990 until 2 June 2021, was conducted by a research librarian (University of Oslo, NO) for this review series in the following databases MEDLINE (Ovid), EMBASE (Ovid) and the Cochrane Central Register of Controlled Trials. The search was registered on the International Prospective Register of Systematic Reviews (PROSPERO ‐ CRD42022276710) where further details are available.^32^ The full electronic search strategy including limits used for the OVID Medline database can be found as Supporting file S2.

The systematic review is part of a comprehensive collaboration including six reviews examining different endpoints in cachexia (body composition, oncology, physical function, PROMs, systemic inflammation, and nutritional). One search was performed for all reviews followed by central appraisal, data extraction and quality assessment. Thereon, eligible trials were reviewed and those specifically examining quality of life were included in the present review. For the present review, the search was updated from 2 June 2021 to 17 October 2023.

### Eligibility criteria

Articles were considered eligible if they were controlled trials investigating interventions which aimed to treat or attenuate cachexia (defined as detailed in PROSPERO) in adults with cancer. There were no restrictions on the type of intervention (pharmacological, nutritional, exercise, multimodal, etc.) or type of comparator. To reduce bias and focus on outcomes with the most clinical impact, trials were excluded if they had randomized fewer than 40 patients, and the intervention lasted <14 days.

For the present review on QOL, some additional inclusion criteria were applied:

- Patient reported QOL (used interchangeably with HRQOL) should be a stated outcome
- Use of validated QOL measures, not ad‐hoc measures
- Studies where QOL partial domains of PROMS (e.g., EORTC emotional functioning) were used were eligible

The following exclusion criteria were applied:

- Insufficient reporting of QOL (i.e., data not shown, not compared between intervention and control groups, or lack of appropriate statistical measures)
- Trials using observer‐rated measures of physical functioning, for example the Karnofsky Performance Status scale (KPS),^33^ or Eastern Cooperative Oncology Group Performance Status scale (ECOG)^34^ as a substitute for self‐reported QOL
- The use of a single symptom scale denoting (e.g., assessing appetite and fatigue) conceptualized as a measure of QOL

### Data selection and extraction

All articles identified were transferred to Covidence software.^35^ Article selection based on titles and abstracts was completed by three researchers in the core team (B. L., T. S .S., and O. F. D.). Any uncertainties in assessing the eligibility of the trials were discussed among the authors until a consensus was reached.

A data extraction table was developed, pilot‐tested and refined within the review group before data were extracted from each article by two independent authors from the review group. Articles relevant to each systematic review were then identified from the data. For this paper, relevant articles assessed the specified QOL endpoints noted in this review.

### Assessing the risk of bias

The methodological quality of each study was systematically assessed by four independent reviewers (J. M. D., J. S., B. L., and O. F. D.) with the Modified Downs & Black Scale.^36^ The measure assesses among other criteria, study design, blinding, sample size, estimate of variance reporting, and whether the outcome is defined and robust.

### Outcomes

This systematic review examines the assessment of QOL in RCTs using validated PROMs on QOL as study endpoints.

More specifically, it describes the following:

- the number of identified cancer cachexia RCTs stating QOL as a primary or secondary, or identified as an exploratory outcome;
- study characteristics and interventions;
- the QOL measures used, including content and properties related to validation, international applicability, mode of administration, dimensionality, scoring methods and interpretation;
- the reporting of QOL results, including statistical methods; and
- correlations with significant primary or secondary study outcomes, as appropriate.

### Data analyses

As expected, the number of retrieved trials was large and heterogeneous. Given this volume and with the main objective being to describe the frequency and diversity of QOL endpoints used, a meta‐analysis of the effect of the interventions was not relevant. Hence, the data were summarized narratively. In trials reporting significant findings on any QOL parameter, raw scores on these subjective measures and the corresponding variability were extracted (if available) to enhance the interpretation of results.

## Results

### Identified cancer cachexia clinical trials with quality of life as an outcome

**Figure 1:**
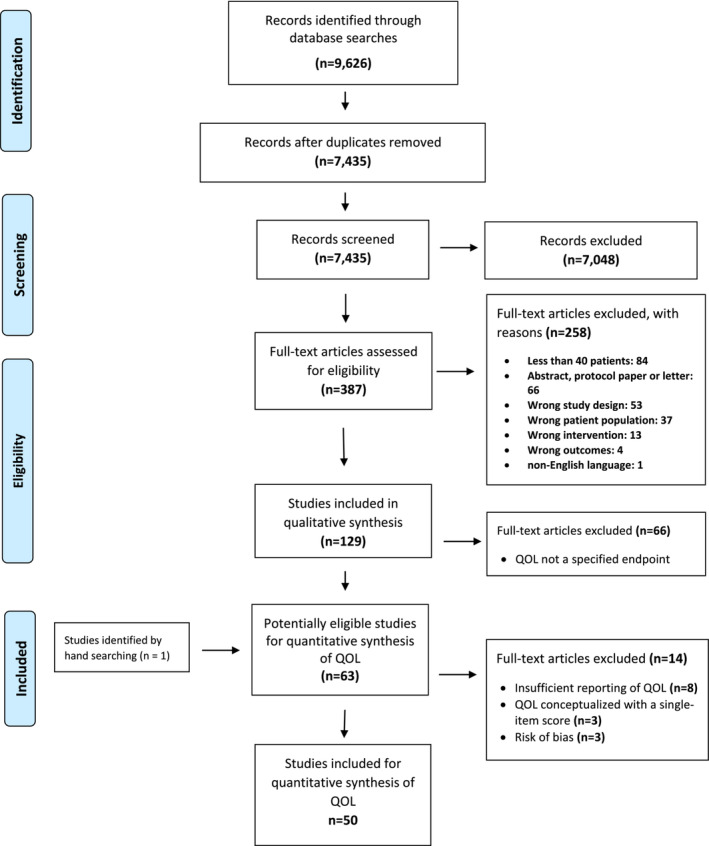
PRISMA flow chart.

The systematic literature search for the series of reviews on cachexia outcomes identified 8166 trials (Figure 1). After deleting duplicates, 7435 papers were retained to screen abstracts, producing 387 articles for full‐text reading.

### Study characteristics

The characteristics of the 50 included trials with QOL outcomes are reported in Table 1. These were published between 1996 and 2023, and conducted in 20 different countries, most often the United States and China (both = 6) followed by Italy, Australia, and Iran (*n* = 4 for all). Three trials were multinational.^38^, ^50^, ^78^ The total sample size based on the number of randomized patients was 6893, but varied considerably across trials, ranging from 42^42^ to 469.^56^

**Table 1:** Key characteristics of eligible trials

1st author	Publ year	Country	Quality^a^	Design	*N* ^b^	Cancer	Intervention	Comparator	Primary outcome (s)	Secondary outcome (s)	QOL Endpoints
Agteresch^37^	2000	Netherlands	7	Open RCT	58	Lung (NSCLC)	Pharmacological:Adenosine 58‐triphosphate	None	QOLWeight loss/gainAlbuminMuscle strength		Rotterdam Symptom Checklist (RSCL)
Bauer^38^	2005	Multinational	8	Double‐blind RCT	200	Pancreatic	Nutritional: Dietary nutritional intervention (protein and energy dense, n‐3 fatty acid, EPA oral)	Isocaloric, isonitrogenous control supplement without n‐3 fatty acids	QOLDietary intakeBody composition		EORTC QLQ‐C30
Beller^39^	1997	Australia	4	Double‐blind RCT	240	GI, mixedHead and NeckHaematological Lung/Pleura	Pharmacological:Arm 1: MA 480 mg per day Arm 2: MA 160 mg per day	Placebo	QOL composite score (LASA + Spitzer QLI‐Index + combined nutritional status score	Separate QOL scoresNutritional status Survival time	LASA
Bouleuc^40^	2020	France	7	Open RCT	148	BreastGI mixedLungMelanomaPelvisProstateSarcomaOther	Nutritional: Parenteral	Oral feeding	QOL (overall QOL, physical functioning, fatigue)	Other QOL scores Nutritional parametersSurvival	EORTC QLQ‐C15‐PAL
Britton^41^	2019	Australia	8	Stepped‐wedge, cluster, RCT	307	Head and Neck, mixed	Multimodal: Motivational interview cognitive behavioural therapy (Eating As Treatment)	Usual standard care	Nutritional status	QOLDietitian SGA ComplianceRe‐admissionsLength of stayDepression	EORTC QLQ‐C30
Bumrungpert^42^	2018	Thailand	6	Double‐blind RCT	42	BreastGI, mixedLungLymphoma	Nutritional: Whey protein isolates	Placebo (maltodextrin) as a daytime snack	Nutritional statusGlutathione levelsImmunityInflammatory status	QOL (explorative)Symptoms	EORTC QLQ‐ C30
Capozzi^43^	2015	Canada	8	Single‐blinded RCT	60	Head and neck Unknown primary	Multimodal Exercise/Lifestyle: Immediate lifestyle intervention	Delayed lifestyle intervention	Body composition BMI, lean body mass, % body fat	QOLFitnessDepressionNutritional status	FACT‐AnFHNSI‐22
Cereda^44^	2019	Italy	8	Double‐blind RCT	166	BloodBreastGI mixed Head and NeckLung	Nutritional: Nutritional counselling + whey protein isolate supplement	Nutritional counselling	Phase‐angle (3 months)	QOLPhase angle (1 month) Standardized phase angleFat‐free mass indexWeightHGSChemotherapy toxicity	EORTC QLQ‐C30
Chen^45^	2023	China	7	Open RCT	108	GI, mixed	Nutritional:Five‐step intervention, education, diet, enteral nutrition, partial enteral/parenteral nutrition, parenteral nutrition	Standard nutritional care	Nutritional status	QOL	EORTC QLQ‐C30
Currow^46^	2021	Australia	6	Double‐blind RCT (phase III)	190	GI, mixed Lung Prostate	Pharmacological:Arm 1: MA	Arm 2: Dexamethasone Arm 3: Placebo	Appetite score	QOLWeightECOG‐PS/KPS	FACT‐G
Dehghani^47^	2020	Iran	7	Single‐blinded RCT	43	GI, mixed	Pharmacological: Angiotensin‐converting enzyme inhibitor	Placebo starch pills	QOL	Weight	EORTC QLQ‐C30
Del Fabbro^48^	2013	USA	10	Double‐blind RCT (phase III)	73	Advanced cancers mixed,	Pharmacological: Melatonin	Placebo	Appetite score	QOLSymptomsFatigueBody compositionWeight	FACIT‐F13FAACT 12 itemESAS
Famil‐Dardashti^49^	2020	Iran	8	Double‐blind RCT	67	Breast GI, mixed Lung Other	Pharmacological: Herbal combination (Fenugreek, Fennel, Chicory) + MA	Placebo +MA	Weight gain	QOLAnthropometric indexes HGSSymptom burdenAnorexia/cachexia symptoms	EORTC QLQ‐C30 FAACT 12 item ESAS
Fearon^50^	2003	Multinational	8	Double‐blind RCT	200	Pancreatic	Nutritional: Protein and energy dense n‐3 PUFA enriched oral supplement	Oral supplement (without n‐3 PUFAs	WeightBody compositionDietary intake	QOL	EORTC QLQ‐C30 EQ‐5D
Gavazzi^51^	2016	Italy	7	Open RCT	79	GI, mixed	Nutritional: Home enteral nutrition	Nutritional counselling	Nutritional status (weight, biomarkers, muscle strength)	QOL (explorative)Treatment compliance	FAACT 12 item
Hong^52^	2020	China	9	Open RCT	204	Breast GI, mixed	Multimodal Exercise/Lifestyle: Resistance exercise	Relaxation	Physical function	QOL	EORTC QLQ‐C30
Hunter^53^	2021	Egypt	7	Double‐blind RCT(phase III)	120	Breast GI, mixed Lung Pleura Other	Pharmacological: Mirtazapine	Placebo	Appetite score	QOLFatigueDepressive symptomsWeightLean body massHGSOverall survivalCRP, IL‐6, YKL‐40	FAACT 12 itemFACT‐GESAS
Isenring^54^	2004	Australia	8	Open RCT	60	GIHead and Neck	Nutritional: Nutrition intervention	Usual standard care	QOLWeightFoot‐to‐foot bioelectrical impedance Nutritional status		EORTC QLQ‐C30
Izumi^55^	2021	Japan	6	Open RCT	81	GI, mixedBladderCarcinoma, unknown LeukaemiaLungMal. mesotheliomaSoft tissue sarcomaThyroidUrological	Pharmacological: Testosterone enanthate administration	None	QOL	Cancer cachexia‐related biomarkersSurvival	FAACT 12 item ESAS
Jatoi^56^	2002	USA	10	Double‐blind RCT	469	GI, mixedLungOther	Pharmacological: Arm 1: MA liquid suspension 800 mg orally daily + capsule placebosArm 2: Dronabinol capsules 2.5 mg orally twice a day + liquid placeboArm 3: Combination of Arm 1&2 medications and dosage	Across arms	AppetiteWeight gain	QOL Toxicity data	FAACTSpitzer QOL index
Jatoi^57^	2010	USA	7	Double‐blind RCT	61	Lung (NSCLC)	Pharmacological: Infliximab + docetaxel	Placebo + docetaxel	Weight gain	QOL (explorative)Appetite changesTumour response rates	FACT‐G
Jatoi^58^	2017	USA	8	Double‐blind RCT	263	GI, mixed LungOther	Nutritional:Creatine	Placebo	Weight gain	QOLWeight stabilityAppetite changesHGSBioelectrical impedance	FAACT 12 item LASA scales
Kanat^59^	2013	Turkey	8	Open RCT	62	Breast GI mixedLung Urogenital Other	Pharmacological:Arm 1: MA + meloxicamArm 2: MA + meloxicam + oral eicosapentaenoic acid‐enriched nutritional supplementArm 3: Meloxicam + oral eicosapentaenoic acid‐enriched nutritional supplement	Comparisons across arms	Weight Lean body mass	QOLBMIIL‐6, TNF‐α	FAACT 12 itemVAS (0–100 for appetite)
Katakami^60^	2018	Japan	8	Double‐blind RCT	174	Lung (NSCLC)	Pharmacological: Anamorelin	Placebo	Lean body mass	QOLWeightBody compositionAppetiteFatigue scoreECOG‐PS/KPS HGS 6‐minute walk testBiomarkers	QOL‐ACD
Kouchaki^61^	2018	Iran	8	Double‐blind RCT (phase III)	90	GI, mixed	Pharmacological: MA + celecoxib	MA + placebo	Weight	QOLHGSAppetite scoreECOG ‐PS Plasma albuminCRP, IL‐6Glasgow Prognostic Score	EORTC QLQ‐C30
Maccio^62^	2012	Italy	8	Open RCT (phase III)	144	Gynaecological, mixed	Pharmacological: Antioxidant agents + L‐carnitine + celecoxib + MA	MA	QOLLean body massResting energy expenditureFatigue	AppetiteGrip strengthGlasgow Prognostic ScorePerformance statusCRP, IL‐6, TNF‐a	EORTC QLQ‐C30
Mantovani^63^	2010	Italy	7	Open RCT (phase III)	332	BreastGI, mixedGynaecological Head and neckLungUrogenital	PharmacologicalArm 1: MPA (500 mg/day) or MA (320 mg/day)Arm 2: EPA‐enriched (2.2 g/day) ProSure and Resource Support or 3 Forticare cartons/day Arm 3: L‐carnitine 4 g/dayArm 4: Thalidomide 200 mg/day Arm 5: MPA or MA plus EPA‐enriched nutritional supplement + L‐carnitine plus thalidomide	Comparisons across arms	Lean body massResting energy expenditureFatigue	QOLAppetiteGrip strengthGlasgow prognostic scoreProinflammatory cytokines	EORTC QLQ‐C30EQ‐5D index/VAS
McMillan^64^	1999	UK	7	Double‐blind RCT	73	GI, mixed	Pharmacological: MA + ibuprofen	MA + placebo	QOLWeight gain	AlbuminCRP	EORTC QLQ‐C30 EQ‐5D
Mehrzad^65^	2016	Iran	8	Double‐blind RCT	70	Advanced cancer, mixed,	Pharmacological: Pentoxifylline	Placebo	Weight loss/gainArm circumstance	QOL	SF‐36
Meng^66^	2021	China	8	Open RCT	353	GI, mixed	Nutritional: Post‐discharge oral nutritional supplements (ONS) with dietary advice	Dietary advice	Nutritional outcomes (BMI,SMI) Sarcopenia prevalence	QOL Chemotherapy tolerance 90‐day readmission rate	EORTC QLQ‐C30
Navari^67^	2010	USA	7	Open RCT	80	ColonLung	Pharmacological:MA + Olanzapine	MA	Weight gainAppetite stimulation	QOLNausea	FACT‐GMDASI
Obling^68^	2019	Denmark	7	Open RCT	47	GI, mixed	Nutritional: Dietic counselling, supplemental home parenteral nutrition	Best practice nutritional care and dietetic counselling	Fat‐free mass	QOLHGSSix minute walk testSkinfold thicknessOverall survival	EORTC QLQ‐C15‐PAL
Persson^69^	2002	Sweden	6	Open RCT	142	GI, mixed	Multimodal: Arm 1: Individual nutritional support Arm 2: Group rehabilitationArm 3: Individual support + group rehabilitation	Arm 4: Usual standard care	QOLWeight changes Food intakeSurvival		EORTC QLQ‐C30
Poulsen^70^	2014	Denmark	5	Open RCT	61	GI, mixed Gynaecological	Nutritional: Nutritional counselling High‐protein nutrition supplement with 3‐fatty acids	Nutritional advice nurses or dieticians	Weight‐loss % weight gain	QOLTreatment related side effects	EORTC QLQ‐C30
Qiu^71^	2020	China	6	Open RCT	96	Oesophageal	Nutritional: Whole‐course nutritional management by nutrition support team	Nutritional supplements (protein, fat, carbohydrate, dietary fibre, minerals, vitamins)	PrognosisChemoradiotherapy complications	QOLNutritional statusIncidence of complications	EORTC QLQ‐C30
Ravasco^72^	2005	Portugal	7	Open RCT	75	Head and Neck	Multimodal: Arm 1: Dietary counselling with regular foodsArm 2: Usual diet plus supplements	Maintained intake ad lib.	Weight	QOLNutritional intake	EORTC QLQ‐C30
Rowland^73^	1996	USA	10	Double‐blind RCT	243	Lung (SCLC)	Pharmacological:MA	Placebo	Survival	QOL (explorative)Response rateWeightToxicity	Spitzer QOL index
Silander^74^	2011	Sweden	6	Open RCT	134	Head and NeckUnknown primary	Nutritional:PEG before start of treatment and individual nutritional support	Usual standard care	Malnutrition	QOLHospital stay	EORTC QLQ‐C30QLQ‐H&N35
Sim^75^	2022	Korea	8	Open RCT	58	GI, mixed	Nutritional: ONS enriched with omega‐3 fatty acids	Standard nutritional care	Nutritional status	QOL Cytokine levels	EORTC QLQ‐C30
Simons^76^	1996	Netherlands	7	Double‐blind RCT	206	GI, mixedLung (NSCLC)Other	Pharmacological: Medroxyprogesterone acetate	Placebo	AppetiteWeight	QOLSide effects	EORTC‐QQL‐C30
Storck^77^	2020	Switzerland	10	Open RCT	52	BreastGI, mixed OvarianLung Urothelial	Multimodal: Leucine‐rich supplement combined with nutritional counselling and physical exercise program	Standard care	Physical function	QOLNutritional statusDietary intakeFatigueCRP	EORTC QLQ‐C30
Strasser^78^	2006	Multinational	8	Double‐blind RCT (phase III)	243	GI, mixedHead and NeckHematologic‐lymphogenicLungUrogenitalOther	Pharmacological:Arm 1: Cannabis extract Arm 2: Delta‐9‐tetrahydrocannabinol	Arm 3: Placebo	QOLAppetite score		EORTC‐QLQ C30
Takayama^79^	2016	Japan	8	Double‐blind RCT (phase II)	181	Lung (NSCLC)	Pharmacological:Arm 1: Anamorelin 50 mgArm 2: Anamorelin 100 mg	Arm 3: Placebo	Lean body massHGS	QOLBody composition WeightSymptomsECOG‐PSKPS Serum biomarkers	QOL‐ACD
Uster^80^	2018	Switzerland	9	Open RCT	58	GI, mixedLung Other	Multimodal: Standardized individual nutritional counselling + exercise program	Usual standard care	QOL (overall QOL)	Dietary intakeNutritional statusPhysical function tests (HGS, lower limb strength, walking capacity, maximal muscle strength)Performance status	EORTC QLQ‐C30
Van der Werf^81^	2020	Netherlands	9	Single blinded RCT	107	GI metastatic, mixed	Nutritional: Nutritional counselling Encouragement of physical activity	Standard care	Muscle mass	QOL Weight Muscle density Hand grip strength Treatment toxicity, intensity, response Progression free overall survival	EORTC QLQ‐C30
Wen^82^	2012	China	5	Open RCT	108	BreastGI, mixedLung	Pharmacological:MA + Thalidomide	MA	QOLWeightFatigue	AppetiteGrip strengthIL‐6 or TNF‐αGlasgow prognostic scorePerformance status	EORTC QLQ‐C30
Westman^83^	1999	Sweden	7	Double‐blind RCT	255	BreastGI mixedGynaecological Head and neckHepatocellularLeiomyosarcomaLung LymphomaMesothelioma MelanomaUrogenital	Pharmacological: MA	Placebo	QOL	SurvivalWeightMA side‐effects	EORTC QLQ‐C30
Wiedenmann^84^	2008	Germany	7	Double‐blind RCT (phase II)	89	Pancreatic	Pharmacological: Infliximab	Placebo	Lean body mass	QOLOverall + progression free survivalKPS6‐minute walk test FatigueNutritional healthPainPhysical + mental functioningTNF‐alpha, CRP, IL‐6, IL‐2	FACIT–F13FAACT SF‐36
Woo^85^	2016	Korea	9	Double‐blind RCT (phase III)	67	Pancreatic	Pharmacological: Pancreatic Exocrine Replacement Therapy Pancreatine‐digestive enzymes (proteins)	Placebo	Weight	QOLPG‐SGA scoreDietary intake Abdominal painFlatulenceOverall survival	EORTC QLQ‐C30
Xie^86^	2018	China	8	Double‐blind RCT	54	Lung (NSCLC)	Pharmacological: Thalidomide and cinobufagin	Cinobufagin	QOLNutritional statusSide effects		EORTC QLQ‐C30

^a^ By the Downs & Black checklist.

^b^ No. of patients randomized.

ECOG‐PS, European Cooperative Oncology Group Performance Status; EORTC QLQ‐C30, European Organization for Research and Treatment Quality of Life Questionnaire; EORTC QLQ‐C15‐PAL, European Organization for Research and Treatment Quality of Life Palliative Care; EORTC QLQ‐C30 QLQ‐H&N35, EORTC QLQ‐C30 QLQ head and neck module; EPA, eicosapentaenoic acid; EQ‐5D, EuroQoL 5D‐Health‐Related Quality Of Life; ESAS, Edmonton Symptom Assessment System; FACT‐An, Functional Assessment of Cancer Therapy – Anaemia scale; FACT‐G, Functional Assessment of Cancer Therapy ‐ General; FAACT 12 item, Functional Assessment of Anorexia/Cachexia Treatment 12 item version; FHNSI‐22, FACT Head/Neck Symptom Index‐22; FACT‐G, Functional Assessment of Cancer Therapy ‐ General; FACIT‐F13, Functional Assessment of Cancer Therapy; GI, Gastro intestinal; HGS, Hand‐grip strength; KPS, Karnofsky Performance Status; LASA, Linear analogue Self‐Assessment scales; MA, Megestrol acetate; MDASI, M.D. Anderson Symptom Inventory MPA, Medroxyprogesterone acetate; PEG, Percutaneous endoscopic gastrostomy; PG‐SGA, patient‐generated subjective global assessment; QOL‐ACD, Quality of Life Questionnaire for Cancer Patients Treated with Anti‐Cancer Drugs; QOL, Quality of Life; SF‐36, MOS short‐form 36‐survey; WL, weight loss.

Most trials (39/50, 78%) included multiple diagnostic groups. Thirty‐one trials mentioned all cancer diagnoses involved, while eight were less specific using broad terms such as gastrointestinal or advanced cancer (Table 1). Twenty‐five of the 50 trials (50%) included patients with lung cancer while pancreatic cancer (42%) was the second most common diagnostic group; Lung (*n* = 6) and pancreatic cancers (*n* = 4) were the two diagnoses most used in the trials limited to one cancer type (Table 1).

#### The interventions

Pharmacological interventions dominated (27/50, 54%) with diverse pharmacological agents, that is, anticancer drugs, appetite stimulants, anti‐inflammatory drugs, and dietary supplements (Table 1). Seventeen trials (34%) were categorized as nutritional interventions, and composed with different nutritional agents, and dietary counselling. Nutritional interventions were, for example, whole‐course nutritional management programme provided by a specialized or multiprofessional teams,^45^, ^71^ protein and energy‐dense oral nutritional supplement with n‐3 fatty acids,^38^, ^75^ whey protein isolate supplements,^44^ or thorough follow up of nutritional status with tube feeding or parenteral nutrition as necessary.^74^, ^81^ Nutritional advice was also included in some of the six multimodal programmes, for example, the cognitive behavioural intervention by Britton et al.^41^ and combined with physical exercise.^77^, ^80^ The median duration of the interventions was 12 weeks (range 4–96).

### Study outcomes

#### Trials with quality of life as the primary outcome

Fifteen trials (30%) had QOL as the primary study outcome.^37^, ^38^, ^39^, ^40^, ^47^, ^54^, ^55^, ^62^, ^64^, ^69^, ^78^, ^80^, ^82^, ^83^, ^86^ QOL was the single primary outcome in six of these trials^39^, ^40^, ^47^, ^55^, ^80^, ^83^ and one of three or four co‐primary outcomes in the remaining nine (Table 1). Five of the 15 trials (27%) defined a clinically meaningful change for QOL, either as a 5 or 10% change in the scales or item scores^38^, ^40^, ^80^ or specified as a difference of .45 or .5 SD.^39^, ^83^ All except four^37^, ^39^, ^40^, ^55^ of these 15 trials used the EORTC QLQ‐C30, either alone (*n* = 7) or in combination (*n* = 4) with other PROMs (Table 1).

The reporting of QOL results varied. Mean (standard [SD]) scores with corresponding p‐values for patient groups were used in eight trials.^47^, ^54^, ^62^, ^64^, ^78^, ^80^, ^83^, ^86^ Two trials reported mean (standard error of the mean [SEM]) or mean (95% confidence interval [CI]) values^38^, ^54^ and one presented the median and range of scores.^69^ Four trials^39^, ^40^, ^55^, ^82^ reported the absolute or per cent change in mean scores at the different assessment points, while one study presented both mean (SD) and per cent change.^39^

#### Trials with quality of life as secondary or exploratory outcomes

Thirty‐one trials (62%) used QOL as a secondary outcome.^41^, ^43^, ^44^, ^46^, ^48^, ^49^, ^50^, ^52^, ^53^, ^58^, ^59^, ^60^, ^61^, ^65^, ^67^, ^68^, ^70^, ^71^, ^72^, ^76^, ^77^, ^79^, ^84^, ^85^ Four of these specified a clinically significant difference for the QOL measures, being a 10%, 20% or 25% difference on the 0–100 scales between groups. This difference was assessed either at a specific assessment point or as a within‐group change over time.^49^, ^61^, ^72^, ^74^

Sometimes QOL measures were not specified as a study objective even if the QOL results were presented in the results section. However, if the latter applied these data were assessed and we defined QOL as an exploratory outcome in four trials.^42^, ^51^, ^57^, ^73^ None of these trials defined a clinically significant difference for the QOL measures.

### The quality of life measures

**Figure 2:**
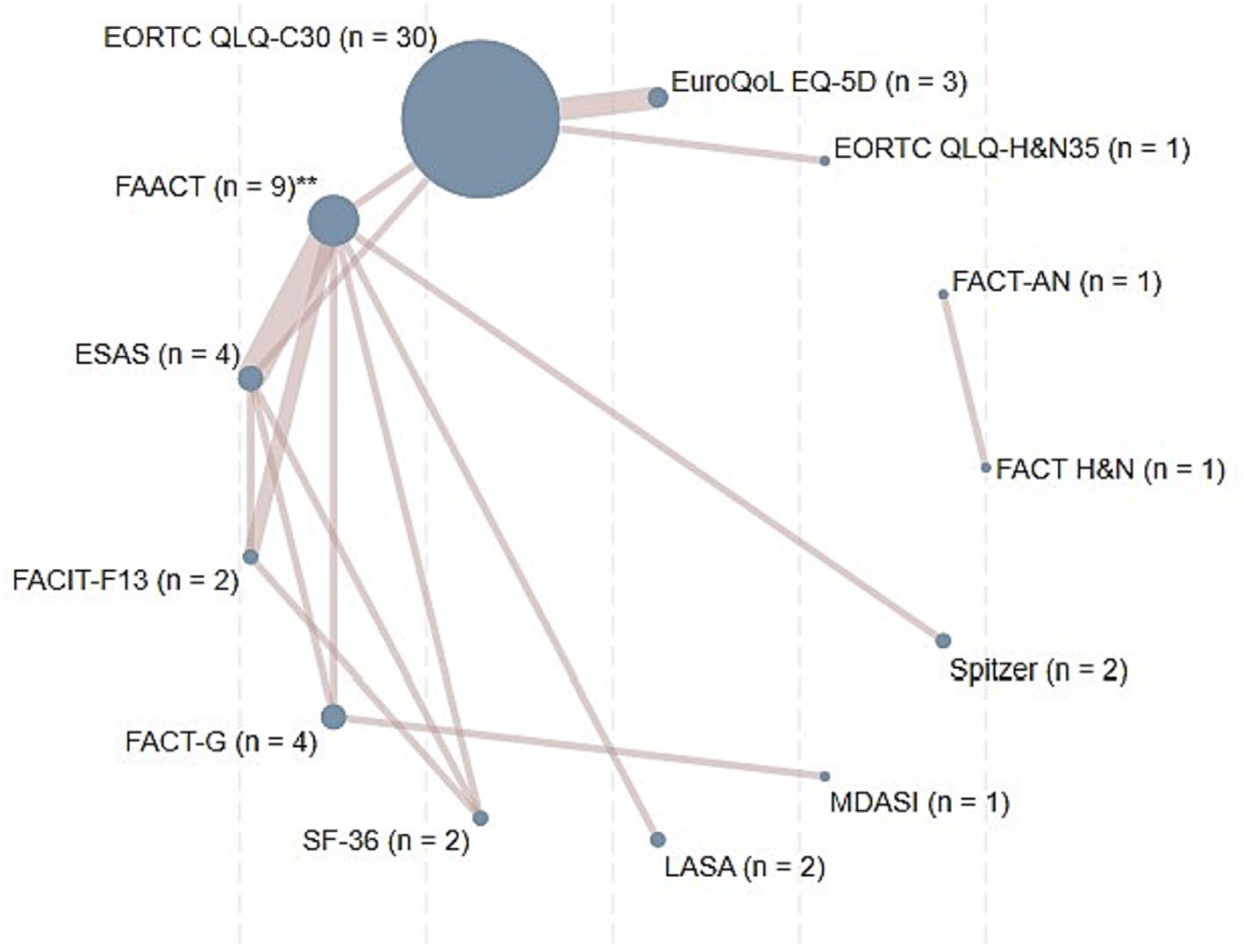
Network diagram reporting of QOL measures. The size of the circles represents the frequency of each measure, and the width of the connecting lines reflects the number of studies reporting each pair of measures. The measures QOL‐ACD, RSCL, and EORTC QLQ‐C15‐PAL are not shown as these have not been presented in combination with other measures of QOL. **FAACT includes both the 12‐ and 18‐item version of this measure. Numerical details are reported in Table 1.

Seventeen different QOL measures were used in these 50 trials. Two of these, the SF‐36^18^ and the EQ‐5D^19^ are generic QOL measures while the remaining 15 are cancer‐specific. The most commonly used measure was the EORTC QLQ‐C30^21^ in 60% of the trials, while 17 trials (34%) used different versions of the FACIT. Thirteen trials (26%) used multiple measures of QOL, often including diagnosis or condition specific measures, such as the EORTC H&N35^25^ and the anaemia and fatigue FACIT measures.^27^ FAACT was the only cachexia‐specific measure used, either in the 18‐ or 12‐Item versions.^29^ Supporting file S3 presents the measures used, their content, assessment period, scoring, number of scales and items and whether a summary measure could be calculated. Figure 2 indicates how often different measures were reported together. With the exception of the measure developed in Japan by Kurihara et al.,^24^ all measures were validated in an international context and demonstrated cross‐cultural applicability.

#### Trials reporting statistically significant quality of life results

Eighteen trials reported statistically significant QOL benefits in favour of the intervention arm.^37^, ^39^, ^41^, ^45^, ^49^, ^52^, ^54^, ^56^, ^60^, ^62^, ^64^, ^66^, ^67^, ^68^, ^74^, ^75^, ^79^, ^82^ Nine of these 18 studies (50%) used pharmacological interventions, and had a total sample size of 2895 (ranging from 47 to 469). The EORTC QLQ‐C30 was the most common measure; used in 61% (11/18) of the trials. The length of the intervention in these trials varied from eight to 28 weeks. Two trials had a pre‐set definition of a clinically significant difference, that is, a difference of 10 points or more on the EORTC‐QLQ‐C30^52^, ^74^ and on the QLQ‐H&N35.^74^

QOL was the primary outcome in six (32%) of these trials^37^, ^39^, ^54^, ^62^, ^64^, ^82^ and a secondary outcome in 12.^41^, ^45^, ^49^, ^52^, ^56^, ^60^, ^66^, ^67^, ^68^, ^74^, ^75^, ^79^ Authors' interpretation of the QOL results are summarized in Table 2, with the statistical presentation of significant results in Table 3. None of these 18 trials reported statistical correlations between the QOL outcomes and other outcome measures. If a potential relationship was mentioned, this appeared in the discussion section and was vaguely described as ‘*being associated with*’ symptom items or other from the intervention endpoints, for example weight gain in the questionnaires.

**Table 2:** Studies reporting significant QOL results

Studies with QOL as their primary outcome (*N* = 6)											
1st author	Publ year	Sample size^a^	Type of intervention	Duration of intervention	Assessment points	QOL measure(s)	Use of QOL measure(s)	Use of other PROM	Significant results^b^	Effect measures^c^	Authors' interpretation
Agteresch^37^	2000	58	Pharma	Max 10 infusions (2–4 week intervals)	Week 0, 4, 8, 12, 16, 20, 24, 28	RSCL	Complete measure	‐	Less decline in PF, functional/psychologic state, overall QOL, sustainable over 4‐week periods	Mean (SD), log‐rank test, GEE^4^	Marked beneficial effect of ATP on QOL
Beller^39^	1997	240	Pharma	12 weeks	Week 0, 4, 8, 12	LASA Spitzer (physician rated)	Complete measure	‐	Better appetite, mood, Overall QOL	Mean (SD), log‐rank test, Cox regression, GEE^4^	Patient‐reports disclose important QOL dimensions, not captured by physician rating
Isenring^54^	2004	60	Nutritional	12 weeks	Week 0, 4, 8, 12	EORTC QLQ‐C30	Global QOL scale, PF	‐	Better QOL and PF in control group	Mean (SEM) χ2, GEE	Less global QOL/PF decline in control group. Weight maintenance may impact PF
Maccio^62^	2012	144	Pharma	4 months	Month 0–4	EORTC QLQ‐C30	Global QOL scale	‐	Greater change in QOL over time	Mean (SD), χ2, Student's t‐/Wilcoxon/rank sum	Multimodal interventions favourable
McMillan^64^	1999	73	Pharma	12 weeks	Week 0, 4–6, 12	EORTC QLQ‐C30 EQ 5D	Complete measures	‐	Weeks 4–6: sign. Better appetite in both groups Week 12: Better QOL (EQ 5D)	Median (range), Mann–Whitney U, Fisher's exact test, Wilcoxon signed rank Freidman test	Weight gain may be associated with better QOL
Wen^82^	2012	108	Pharma	8 weeks	Week 0–8	EORTC QLQ‐C30	Global QOL scale	Appetite (VAS)	Greater change in QOL over time	Mean scores (SD), Mean change (SD) Student's t, χ2	Adequate fat‐free mass may contribute to better QOL

Studies with QOL as a secondary outcome (*n* = 12)											
1st author	Publ year	Sample size^a^	Type of intervention	Duration of intervention	Assessment points	QOL measure(s)	Use of QOL measure(s)	Use of other PROM	Significant results^b^	Effect measures^c^	Authors' interpretation
Britton^41^	2019	307	Nutritional	6 weeks	Week 0, 1 12 post‐RT	EORTC QLQ‐C30	Complete measure	PG‐SGA	Better overall QOL post radiation therapy	Linear mixed model of QOL mean scores over time	Effective interventions in H&N patients during RT
Chen^45^	2023	108	Nutritional	10 weeks	Week 0–10	EORTC QLQ‐C30	Complete measures	PG‐SGA NRS‐2002	Improved PF and SF, reduced fatigue, pain, appetite loss, constipation	Mean (SD), Shapiro–Wilk test, Chi‐square, Fisher exact test, Wilcoxon Sign‐Rank Test, Mann–Whitney U Test	Nutritional intervention improved the nutrition status and showed positive impact on quality of life
Famil‐Dardashti^49^	2020	67	Nutritional	8 weeks	Week 0, 8	EORTC QLQ‐C30, FAACT	FAACT total score Global QOL score	ESAS	Better QOL after 8 weeks	Mean (SD) paired‐sample/and independent t‐test, Mann Whitney u	Reaching QOL improvement needs a longer duration of follow up in comparison with other indices
Hong^52^	2020	204	Multimodal Exercise/Lifestyle: Resistance exercise	12 weeks	Week 0, 12	EORTC QLQ‐C30	Complete measure	‐	Better PF and RF after 12 weeks	Mean (SD) Student t‐test	Interventions is effective on symptoms, PF and QOL during chemotherapy
Jatoi^56^	2002	469	Pharma (three arms)	Median 80 days	Weekly for 4 weeks monthly thereafter	UNISCALE FAACT‐AN	FAACT‐AN total score	‐	Greater improvement in QOL over time	Independent sample t‐test, Wilcoxon rank sum tests	Better QOL may reflect the emphasis on anorexia of the FAACT‐AN
Katakami^60^	2018	174	Pharma	12 weeks	Week 1, 3, 6, 9, 12	QOL‐ACD	QOL‐ACD scores	‐	Better QOL‐ACD scores on PF, meals, appetite, weight loss	Mean (SD) Least square means (+/‐SD) difference from baseline	Interventions are effective on multiple outcomes in NSCLC patients with CC
Meng^66^	2021	353	Nutritional	3 months	Month 0–3	EORTC QLQ‐C30	Complete measure	‐	Lower weight loss, less sarcopenia, and chemo‐therapy modifications, less fatigue, and appetite loss	Mean (SD), independent‐samples t test, Mann–Whitney U test, χ2, Fisher exact test	Post‐discharge ONS and dietary advice improved nutritional outcomes, skeletal muscle maintenance, chemotherapy tolerance and some QOL variables
Navari^46^	2010	80	Pharma	8 weeks	Week 0, 4, 8	FACT‐G	Complete measure	MDASI	Better QOL at 4 and 8 weeks	Percentage of patients with improvement	Interventions are effective on multiple outcomes in cachectic patients
Obling^69^	2019	47	Nutritional	24 weeks	Week 0, 6, 12, 18, 24	EORTC QLQ‐C15‐PAL	Global QOL scale	‐	Better QOL at week 12, then stabilized	Mean (SD) difference	Home parenteral nutrition may be feasible in select patients
Silander^74^	2012	134	Nutritional Percutaneous Endoscopic Gastrostomy (PEG)	NA	Month 0, 3, 6, 12, 24	EORTC QLQ‐C30 QLQ‐H&N35	Complete measures	‐	Better overall QOL, PF, SF and CF at 6 months	Mean, Mann–Whitney U test	Prophylactic PEG reduced malnutrition improved QOL
Sim^75^	2022	58	Nutritional	3 months	Month 0–3	EORTC QLQ‐C30	Complete measure	PG‐SGA	Worsening of symptoms in control group, Better better Global QOL, RF, sleep, fatigue constipation at week 8	Mean (SD), independent‐samples t test, Mann–Whitney U test, χ2, Fisher exact test	Intervention improves PG‐SGA scores and QOL during chemotherapy
Takayama^79^	2016	181	Pharma	12 weeks	Week 0, 4, 8, 12	QOL‐ACD	Complete measure	MDASI	Greater improvement in QOL over time	Mean (SD) Least squares (LS) mean change from baseline	Promising results on multiple outcomes, especially QOL

^a^ Number of randomized patients.

^b^ Defined as a statistically significant difference across groups or within groups over time. Significance is in favour of the intervention group, unless otherwise stated.

^c^ Statistics used for QOL scores.

CF, cognitive functioning (function scale EORTC QLQ‐C30); GEE, generalized estimating equation; PG‐SGA, patient‐generated subjective global assessment; PF, physical functioning (function scale EORTC QLQ‐C30); RF, role functioning (function scale EORTC QLQ‐C30); RT, radiation therapy; SD, standard deviation; SF, social functioning (function scale EORTC QLQ‐C30).

**Table 3:** Statistical presentation of significant QOL results^a^

Studies with QOL as a PRIMARY outcome (*n* = 6)					
1st author	*P*‐value, difference between‐groups^b^ ^,^ ^c^	Control baseline (mean ± SD) (median + ICR/range)	Control endpoint (mean ± SD) (median + ICR/range)	Intervention baseline (mean ± SD) (median + ICR/range)	Intervention endpoint (mean ± SD) (median + ICR/range)
Agteresch^37^	RSCL Physical QOL; 0.0002RSCL Functional QOL; 0.02RSCL Overall QOL; 0.001	77.9 ± 13.9	Not reported	78.1 ± 12.3	Changes in scores, intervention vs. controlsPhysical (−0.2% vs. − 2.4%; Functional + 0.4% vs. −5.5Overall QOL + 0.1% vs. −3.5%
Beller^39^	LASAP for trend: <0.001Mood: 0.001 Appetite: 0.001Overall QOL: <0.001QOL categorized: <0.001	Average difference in scores between baseline and subsequent weeks 4, 8, 12 Mood; placebo −4.1, low: 0.4, high: 10.9 Appetite: placebo: 9.7, low: 17.0, high: 31.3 Overall QOL: placebo: −2.1, low: 2.4, high: 12.3QOL categorized: placebo: −2.37, low: 2.66, high: 3.05			
Isenring^54^	EORTC QLQ‐C30, global QOL: 0.009	75.3 ± 19.2	62.6 (SEM in Figure)	67.7 ± 18.8	72.7 (SEM in Figure)
Maccio^62^	EORTC QLQ‐C30, global QOL: 0.042	57.0 ± 12.8	61.1 ± 15.5	53.8 ± 17.4	61.3 ± 20.9
McMillan^64^	EORTC QLQ‐C30: NSEQ 5D: <0.05	QLQ‐30: 33.3 (0–91.7)EQ‐5D: 0.630 (−0.095–1.000)	Not reported	EORTC QLQ‐30:33.3 (0–83.3)EQ‐5D: 0.689 (−0.261–1.000)	Not reported
Wen^82^	EORTC QLQ‐C30, within group over timeIntervention, Global score: 0.02Control, appetite: 0.02Between groups: Global QOL: <0.01	50.3 ± 16.6	51.4 ± 19.7	49.0 ± 23.2	56.9 ± 26.3

Studies with QOL as a secondary outcome (*n* = 12)					
1st author	*P*‐value, difference between‐groups^2^ ^,^ ^3^	Control baseline (mean ± SD) (median + ICR/range)	Control endpoint (mean ± SD) median + ICR/range)	Intervention baseline (mean ± SD) (median + ICR/range)	Intervention endpoint (mean ± SD) (median + ICR/range)
Britton^41^	EORTC QLQ‐C30:Global QOL < 0.01CF < 0.01PF 0.01Nausea/vomiting: <0.01Appetite loss: 0.02	Mean EORTC QLQ‐C30 scores from a linear mixed model tabulated across end of treatment, 1 month post‐RT, and 3 months post RT			
Chen^45^	EORTC QLQ‐C30 Global QOL: <0.001 SF: 0.0023 Fatigue: 0.023 Pain: 0.0127 Appetite: 0.0228 Constipation: 0.0004	Global QOL: 62.7 ± 10.1 SF: 83.3 ± 16.4 Fatigue: 7.5 ± 9.1 Pain: 18.1 ± 10.7 Appetite: 18.3 ± 11.8 Constipation: 17.3 ± 12.2	Global QOL: 48.7 ± 15.9 SF: 57.3 ± 15.6 Fatigue: 55.4 ± 21.2 Pain: 61.8 ± 20.1 Appetite: 47.7 ± 14.4 Constipation: 50.7 ± 12.3	Global QOL: 60.5 ± 10.5 SF: 92.1 ± 17.7 Fatigue: 10.2 ± 6.3 Pain: 18.2 ± 15.1 Appetite: 19.8 ± 15.6 Constipation: 16.2 ± 10.3	Global QOL: 66.6 ± 18.1 SF: 88.5 ± 19.6 Fatigue: 30.5 ± 14.8 Pain: 49.1 ± 15.8 Appetite: 22.2 ± 15.4 Constipation: 19.1 ± 9.7
Famil‐Dardashti^49^	EORTC QLQ‐C30 Global QOL: 0.001FAACT Global score: 0.05	64.4 ± 4.9	Not reported	66.9 ± 7.7	Not reported
Hong^67^	EORTC QLQ‐C30 PF: 0.035 RF: 0.041 Fatigue: 0.024	Global QOL: 58.6 ± 20.3PF: 84.7 ± 18.2RF: 64.1 ± 21.3	Global QOL:55.2 ± 22.6PF: 72.4 ± 15.9RF: 54.9 ± 17.9	Global QOL:56.8 ± 19.5PF:83.6 ± 15.7RF:62.3 ± 24.7	Global QOL 60.3 ± 21.7PF: 86.3 ± 17.4RF: 66.7 ± 19.4
Jatoi^56^	FAACTFAACT‐AN QOL: 0.002Dronabinol+MA vs. MA: 0.003FAACT Appetite: <.001FAACT QOL: .009Dronabinol vs MA: 0.003	Megestrol acetate55 (26–84)	Results visualized in Figure only	Dronabinol: 56 (27–92)Megestrol Acetat + Dronabinol:57 (27–92)	Difference between megestrol acetate and dronabinol groups median, 7.8 [range, 0 to 41] v 2.6 [range, 0 to 59]
Katakami^60^	QOL‐ACDPF: 0.017Enjoying meals: 0.032Appetite: 0.0029Total score: NS	70.9 ± 13.0	Least square means ± SD shown in figures every 3 months	74.9 ± 13.0	Least square means ± SD shown in figures every 3 months
Meng^66^	EORTC QLQ‐C30Global QOL: 0.256Fatigue: 0.035Appetite loss: 0.013	Not reported	Global QOL: 73 (50–100)Fatigue: 22 (0–44)Appetite: 8 (0–33)	Not reported	Global QOL: 75 (58–100)Fatigue: 11 (0–33)Appetite: 0 (0–33)
Navari^67^	MDASI summary score:<0.01	Number of patients with improvement in figures, significant value reported in text			
Obling^68^	EORTC QLQ C15‐PAL‐15: Global QOL: <0.05 (after 12 weeks)NS (24 weeks)	Global QOL: 64 ± 17	Global QOL: 56 ± 18	Global QOL: 60 ± 23	Global QOL: 69 ± 24
Silander^74^	EORTC QLQ‐C30Global QOL 0.02 (6 months)(NS 3, 12, 24 months)	Global QOL: 63 (SD not reported)	Global QOL: 52 (SD not reported)	Global QOL: 64 (SD not reported)	Global QOL: 77 (SD not reported)
Sim^75^	EORTC QLQ‐C30 Fatigue: 0.020	Fatigue: 18.52 ± 4.67	Fatigue: 35.21 ± 8.15	Fatigue: 28.28 ± 6.15	Fatigue: 16.66 ± 4.18
Takayama^79^	QOL ‐ACD 0.05 (100 mg vs. placebo) NS (50 mg vs. placebo)	73.4 ± 13.9	Least square means ± SD shown in figures every 4 weeks	50 mg anamorelin: 71.3 ± 14.9100 mg: 70.6 ± 13.9	Least square means ± SD shown in figures every 4 weeks

^a^ Number of randomized patients.

^b^ Defined as a statistically significant difference across groups or within groups over time. Significance is in favour of the intervention group, unless otherwise stated.

^c^ Statistics used for QOL scores.

CF = Cognitive functioning (Function scale EORTC QLQ‐C30), GEE = Generalized estimating equation, PG‐SGA = Patient Generated Subjective Global Assessment, PF = Physical functioning (Function scale EORTC QLQ‐C30), RF = Role functioning (Function scale EORTC QLQ‐C30), RT = Radiation therapy, SD = Standard deviation, SF = Social functioning, (Function scale EORTC QLQ‐C30)

Only three of the 18 trials presenting significant results had defined a magnitude of a statistically and/or clinically significant difference, that is, a 0.45 SD corresponding to an 11% change on the 0–100 overall LASA or Uniscale scores^39^ or a difference of 10 points or more on the EORTC‐QLQ‐C30 measures.^52^, ^74^ None of the trials reported adjustments for multiple testing in the statistical significance analyses, even if most QOL measures were composed of several items and domains.

## Discussion

This review identified 50 RCTs in cancer cachexia where QOL was assessed as an outcome. Overall, 18 trials reported statistically significant differences in QOL outcomes, in favour of the intervention groups. Of these, six had QOL as the primary study outcome, and 12 had it as a secondary outcome. These findings, although encouraging, indicate many considerations are needed when incorporating QOL in cachexia clinical trials.

Firstly, defining what a clinically significant difference represents is challenging, and this was seldom reported. Only one trial (QOL was the primary outcome) defined a clinically meaningful difference in QOL (11%/0.5 SD),^39^ while another used a 10% difference on the 0–100 numerical scales, but did not specify which of the multiple outcomes this applied to.^82^ A ‘rule of thumb’ is that a difference of 7–15% on the 0–100 scale, or a 0.5 SD is meaningful to patients.^87^, ^88^ However, the difference between minimally clinically important differences (MCID) at a patient level versus at the group level is not clear. The latter relates to mean differences between groups or mean change over time reaching a level of significant difference, whereas individual patient change over time categorize, for example, non‐responders/responders to a particular treatment effect is the focus at the patient level. These approaches require different thresholds for correct interpretations as emphasized in ongoing international projects aiming to standardize the measurement and interpretation of PROMs.^89^, ^90^ As cachexia is a multifactorial syndrome, it is important to understand how changes in QOL relate to changes in other endpoints. For example, does improved QOL correlate with improved physical function and vice versa? An understanding of such relationships is critical both for patient benefit and also to know how pathophysiological changes (and therefore potential mechanisms of action of interventions) relate to changes in endpoint(s). None of the 18 studies where QOL improved examined how this related to other endpoints. This represents an area that should be addressed in future trials.

Secondly, sample size calculations need to be applied when QOL endpoints are assessed, although QOL improved in a proportion of studies, sample size estimations in relation to this was uncommon, as were effect sizes.

Thirdly, the optimal time point for measuring QOL needs to be clarified. Usually, these are assessed over time with multiple QOL assessments, and this was the case for some trials included where for example QOL improved after 12 weeks. This finding could mean that a significantly improved QOL at 4 weeks may be sustainable for the next 2 months as well, that it was a random finding, or maybe that it was not attributable to the intervention per se but to other factors influencing QOL. Yet other factors may impact QOL and as does the expected deterioration in patients with cancer cachexia.^3^

Finally, the complex intervention(s) complicates the interpretation of results as disentangling which affects which outcome, is challenging; particularly with QOL. Yet these multimodal interventions in cachexia trials are recommended in cachexia treatment^91^ and practical guidelines.^3^ Additionally regulatory bodies advocate QOL endpoints, so ways to integrate appropriately and assess QOL in cachexia trials is essential and a research priority. Taken together, much work remains to integrate QOL measures optimally and meaningfully into cancer cachexia clinical trials. Some proposals can be found in Supporting file S4.

As QOL assessment is likely to remain a central tenet of the cachexia trial endpoint spectrum, the question remains as to which measure should be used. We noted that the EORTC QLQ‐C30^21^ was the most frequently used measure (as evidenced in other reviews^4^, ^92^ followed by the FACT‐G^20^ (part of the specific FACT‐modules). These are multidimensional, internationally validated and developed through rigorous and stepwise scientific processes. The EORTC QLQ‐C30 and FACT‐G have been adapted to include cachexia‐specific QOL assessment via the EORTC Cachexia‐24 module^30^ and the 12‐item FACIT cachexia‐specific instrument (FAACT).^29^ Whilst these adaptions are welcome further evaluation of these is needed before they can be recommended as being the preferred QOL assessment; specifically, regarding response to change in patients with cachexia.^4^ It could also be proposed that single items from QOL assessments could be assessed or even single items from multiple assessments combined; yet one of the limitations of such an approach is that when tools are dissected in terms of their component parts, the validity is often questioned, and these tools have usually been developed and assessed as a whole.

It is acknowledged that patients with cancer cachexia are often frail or deteriorate rapidly. Thus, the balance between the need for short measures to reduce patient burden and the need for comprehensive assessments may be challenging. However, technological development with digital measures and computer adaptive testing methods of the EORTC and FACIT measurement systems that tailor the questions to the individual patients, represents a major step forward in the monitoring and follow‐up of patients, in research and clinical practice.

Both the EORTC QLQ‐C30 and the FACT‐G/FAACT have composite scores, calculated as the mean of the combined scale and item scores for the EORTC QLQ‐C30, and by totalling subscale FACT‐G scores. For the EORTC QLQ‐C30; however, the distinction between the Global QOL score and the composite score is important, as the former consists of only two items that combine physical health and the patient perception of overall QOL. Only one reviewed paper used the composite EORTC score, while the Global QOL Score was used as the only outcome measure in two of the nine trials reporting significant QOL results with QOL as a primary outcome^62^, ^82^ and supplemented with the physical functioning score.^54^ This is problematic because the Global QOL scale score may not correspond well to specific item or scale scores, as it does not appear to be sufficiently sensitive. A probable explanation might be a response shift over time in patients with advanced disease: ‘*taken together my situation is not that bad*’, despite reporting a relatively high symptom burden. On the other hand, it is acknowledged that an improvement in one specific scale within a multidimensional QOL measure denotes a generally improved QOL. The well‐validated tools contain both single items and multidimensional scales assessing different aspects of QOL including symptom burden, and possess a reasonable sensitivity and specificity. Thus, a sole focus on symptoms such as appetite or fatigue should not be used alone to indicate a multidimensional construct such as QOL. Thus, trials that used a single item to denote QOL in this review were not included. Also, it is discouraging that associations between QOL outcomes and other more objective results were not emphasized in any of the reviewed trials. Further, trials using weight loss or gain as outcomes face several challenges that were rarely elaborated on in the trials. Examples are the variability in measures (per cent, kilograms, and slopes on the weight curves) and the association with body composition variables that may affect muscle mass, strength, and functioning that may affect specific QOL scores, to which a global score is not sufficiently sensitive.

Removing confounding factors, particularly in the context of a clinical trial where QOL is measured, is worthy of note. Of the 15 trials reporting significant QOL differences across groups, nine were pharmaceutical trials, while the remaining six had a more individualized approach. It can be hypothesized that a direct patient‐centred approach with individual or group follow‐ups in terms of meetings, exercise groups, frequent phone calls, or digital consultations may improve the patients' emotional wellbeing and mental state. However, this ‘attention’ effect would be controlled for, though not blinded against, if there were some sort of active intervention in the control group, as opposed to conventional care. To our knowledge, the direct benefits of being in a study are rarely investigated, but it is likely that being ‘seen’ is a positive factor. In cachexia trials where counselling on nutrition and physical activity are commonplace, therapeutic relationships will develop and these are likely to influence QOL for trial participants. It is also reasonable to assume that other aspects may influence QOL, independent of the intervention being assessed. Examples include psychological distress caused by disease progression, side‐effects from anti‐cancer therapy, or financial worries. Disentangling these facets from a ‘pure’ QOL assessment in a clinical trial is complex and may be challenging to truly understand.

### Strengths and limitations

A key limitation of the review was that the heterogeneity of trial designs, populations studied, and variation in interventions prevented direct comparisons of results or a meta‐analysis. This limitation was partly due to the decision to include trials that assessed QOL in varying hierarchies of endpoints. As such, where QOL was not a primary endpoint, the trial was not powered to conclude on QOL results. This approach was justified to ensure that no important information was missed.

The multifactorial origin of cancer cachexia calls for multimodal interventions, even if it makes it difficult to prove an exact relationship between interventions and outcomes. Related to this is that the use of aggregated data limits a detailed assessment of the relationship between different QOL measures and other study outcomes. It is therefore not possible to draw firm conclusions regarding which measure to use, given the QOL measures are multidimensional and their sensitivity to changes in other outcomes is unknown. We also have to acknowledge that in patients with cachexia, other symptoms related to cancer will be common, in addition to co morbidities, in older patients in particular. Choice and use of QOL instruments within the context of clinical trials must be cognizant of these factors.

We purposefully chose to use a quality assessment tool that was general rather than specific to certain endpoints, and we regard this as an appropriate methodology. Further, we decided to do quality assessments at the initial level by reviewers to limit bias. We believe that the selection of another approach for reporting quality assessment endpoints would have had only a minor influence on our conclusion.

It was challenging to distinguish between studies trying to prevent or treat cachexia per se (involuntary weight loss) versus those trying to treat the symptoms caused by cachexia. There was no uniform definition of cachexia used throughout to the extent that some studies examined cancer anorexia (a component of cachexia syndrome) versus targeting lean mass versus targeting multiple components. Indeed, the lack of uniform trial definition reflects the current status quo of cachexia clinical practice whereby there are several, alternative operational diagnostic criteria (e.g., Fearon definition)^1^ or the Global Leadership Initiative in Malnutrition (GLIM) criteria^93^ each of which has been established by expert consensus alone. From a QOL perspective however, it should be acknowledged that objective changes such as weight gain or improved performance status might contribute to improvement in one or more QOL aspects, even if it does not change the patient's cachectic state. Future work should be clear as to the primary aim of any intervention as potentially the term ‘cancer cachexia’ may be too vague in the context of a clinical trial intervention.

One study strength is the review of trials using PROMs that span more than 30 years of research, coupled with the fact that the most frequently used and well‐validated QOL measures in the reviewed trials date back to the early 1990s, that is, the EORTC QLQ‐C30 and FACT‐G. The EORTC QLQ‐C30 was used in the two oldest trials in this review, published in 1999. Also, the thorough approaches used for evaluating scientific quality, extraction, and appraisal of papers is a major strength. This also applies to the careful registration of relevant variables in a common database for a series of reviews of cachexia trials involving double or triple appraisals for paper retention.

## Conclusions

QOL is an important endpoint in cancer cachexia trials, regardless of whether an improvement is due to direct effects from a specific drug or results from synergistic effects of the multiple components in complex interventions. Thus, it makes sense to include patient‐reported QOL endpoints in cancer clinical trials. As demonstrated in this review, however, comprehensive descriptions of the patient samples and characteristics varied, as did the presentation of statistical considerations related to sample size, power estimations, presentation of results and correlations with other study outcomes, and adjusting for multiple significance testing.

Thus, we call for a more rigorous approach to assessing QOL as an endpoint in cancer cachexia trials, including defining what a MCID is, how QOL relates to mechanism of action of the intervention, other key endpoints (e.g., physical function), and learning from other areas where regulatory approval has been given on the basis of a PROM of QOL. As cancer cachexia has a profound impact on patients' QOL, and as it is a multidimensional construct, we recommend the use of well‐validated comprehensive QOL measures with cachexia‐specific modifiers and advise against using single items as surrogate indices of QOL. Taken together, these will inform future trials and clinical practice.

## Conflict of interest

Eric. J. Roeland has served as a member of the scientific advisory board for Napo Pharmaceuticals, Care4ward, Actimed Therapeutics, and Meter Health in the last 2 years, as a consultant for Veloxis Therapeutics, Aileron, and BYOMass, and as a member of the advisory board for Takeda. He has also served as a member on the data safety monitoring boards for Enzychem Lifesciences Pharmaceutical Company. Barry Laird has served as a member of the scientific advisory board for Actimed and Artelo. He has undertaken consultancy for Faraday, Kyona Kirin, and Grunenthal. Andrew S. J. Coats declares to have received honoraria and/or lecture fees from: Astra Zeneca, Bayer, Boehringer Ingelheim, Edwards, Eli Lilly, Menarini, Novartis, Servier, Vifor, Abbott, Actimed, Cardiac Dimensions, Corvia, CVRx, Enopace, ESN Cleer, Faraday, Impulse Dynamics, Respicardia, and Viatris. Richard Skipworth has received grant funding from Novartis, has been an advisory board member for Helsinn and Faraday Pharamaceuticals, and has provided consultancy work for Helsinn, Actimed Therapeutics, and Avidity Biosciences. Marianne J. Hjermstad, Gunnhild Jakobsen, Jann Arends, Trude R. Balstad, Leo R. Brown, Asta Bye, Olav F. Dajani, Ross D. Dolan, Marie T. Fallon, Christine Greil, Alexandra Grzyb, Stein Kaasa, Lisa Koteng, Anne M. May, James McDonald, Inger Ottestad, Iain Philips, Judith Sayers, Melanie R. Simpson, Tora S. Solheim, Mariana S. Sousa, Lisa H Koteng, and Ola M. Vagnildhaug declare that they have no conflict of interest.

## Supporting information


**Data S1.** Supporting Information.

**Data S2.** Supporting Information.

**Data S3.** Supporting Information.

**Data S4.** Supporting Information.
