# Supplementary material for: Quality of life endpoints in cancer cachexia clinical trials: Systematic review 3 of the cachexia endpoints series
Source: J Cachexia Sarcopenia Muscle. 2024 Mar 29;15(3):794–815. doi: 10.1002/jcsm.13453 (PMC11154790; doi:10.1002/jcsm.13453)
Supplement: Supplementary file 2 — Data S2. Supporting Information. [file JCSM-15-794-s004.docx]

**Supplemental file 1**

**Documentation of literature search**

1. Documentation on the literature search for “What is the optimal endpoint to evaluate effect of interventions aiming to treat cancer cachexia?”

The following databases were searched:

| **Database** | **Number of retrieved references for TRIALS** | **Number of retrieved references for Cohort/** **longitudinal studies** |
| --- | --- | --- |
| Medline (Ovid): | 3812 | 1918 |
| Embase (Ovid): | 2033 | 2031 |
| Cochrane Central Register of Controlled Trials: | 1923 |  |
| Number of references before deduplication: | 8166 | 3949 |
| Number of references after deduplication: | 5998 | 3190 |

The searches described below were done 02 Juni 2021 by Gunn Kleven, Senior librarian at the Library of Medicine and Science, University of Oslo (UiO).

Number of hours spent: 30

**Ovid MEDLINE(R) ALL**1946 to July 01, 2021

Date searched: 02 Juni 20201
Search Strategy:

| **#** | **Searches** | **Results** |
| --- | --- | --- |
| 1 | exp Neoplasms/ or (neoplasm* or cancer* or tumor* or tumour* or oncol* or malign* or carcinom* or adenocarcinom* or adenoma or metasta*).ti,ab,kf. | 4606655 |
| 2 | Cachexia/ or Emaciation/ or Malnutrition/ or Starvation/ or Wasting syndrome/ or Thinness/ or Sarcopenia/ or Anorexia/ or *Weight Loss/ | 63432 |
| 3 | and/1-2 | 9650 |
| 4 | ((cachexia or cachexic or anorexia or anorectic or emaciat* or malnutrition or underweight or starvation* or thiness or leanness or sarcopenia or wasting syndrome* or wasting disease* or weightloss* or ((appetite* or weight) adj2 (loss or loosing or losing))) adj4 (neoplasm* or cancer* or tumor* or tumour* or oncol* or malign* or carcinom* or adenocarcinom* or adenoma or metasta*)).ti,ab,kf. | 7231 |
| 5 | ((cachexia or cachexic or anorexia or anorectic or emaciat* or malnutrition or underweight or starvation* or thiness or leanness or sarcopenia or wasting syndrome* or wasting disease* or weightloss* or ((appetite* or weight) adj2 (loss or loosing or losing))) and (neoplasm* or cancer* or tumor* or tumour* or oncol* or malign* or carcinom* or adenocarcinom* or adenoma or metasta*)).ti. | 4253 |
| 6 | or/3-5 | 13924 |
| 7 | randomized controlled trial.pt. | 536354 |
| 8 | controlled clinical trial.pt. | 94265 |
| 9 | randomized.ab. | 525221 |
| 10 | placebo.ab. | 219320 |
| 11 | drug therapy.fs. | 2343029 |
| 12 | randomly.ab. | 360557 |
| 13 | trial.ab. | 557904 |
| 14 | groups.ab. | 2213680 |
| 15 | or/7-14 | 5047938 |
| 16 | exp animals/ not humans.sh. | 4855037 |
| 17 | 15 not 16 | 4388865 |
| 18 | 6 and 17 | 4078 |
| 19 | limit 18 to yr="1990 -Current" | 3812 |
| 20 | cohort studies/ or follow-up studies/ or longitudinal studies/ or "national longitudinal study of adolescent health"/ or prospective studies/ or retrospective studies/ | 2168707 |
| 21 | (cohort* or longitudinal or prospective* or retrospective*).tw. | 2098508 |
| 22 | or/20-21 | 3012965 |
| 23 | and/6,22 | 3215 |
| 24 | limit 23 to yr="1990 -Current" | 3139 |
| 25 | 24 not 19 | 1918 |
| 26 | 19 or 24 | 5730 |

The same terms were used for the updated search, July 2^nd^, 2023 to October 30^th^, 2023, producing 1,460 new references. Senior librarian Gunn Kleven, UiO performed this search as well
