# Supplementary material for: Quality of life endpoints in cancer cachexia clinical trials: Systematic review 3 of the cachexia endpoints series
Source: J Cachexia Sarcopenia Muscle. 2024 Mar 29;15(3):794–815. doi: 10.1002/jcsm.13453 (PMC11154790; doi:10.1002/jcsm.13453)
Supplement: Supplementary file 3 — Data S3. Supporting Information. [file JCSM-15-794-s003.docx]

**Supplemental file 2. QOL measures used**

| **Measure name** | **TYPE OF INSTRUMENT** | **CONTENTS** | **SCORING METHODS^1^** | **CALCULATIONS** | **TIME FRAME^1^** |
| --- | --- | --- | --- | --- | --- |
| **EORTC QLQ-C30** [21] | - QOL in patients with cancer | Common symptoms in cancer  30 items   - 5 functional scales (physical, role, cognitive, social emotional) - 3 symptom scales (fatigue, pain nausea/vomiting) - 6 single items - 1 Global QOL scale | - 28 symptom items on ordinal scales; 1: *not at all* - 4: *very much* - 2 items on overall health and QOL; 1: *not at all* - 7 *excellent* | - Scores converted to continuous 0-100 scales - Overall score | Past week |
| **EORTC-QLQ-HN35** [25] | - Additional module to the QLQ-C30 for patients with H&N cancer | Common symptoms in H&N cancer  35 items   - 7 therapy / tumour-focused scales (e.g., pain, swallowing, taste) - 6 symptom items (e.g., dental problems, trismus, sticky saliva) | - All items on ordinal scales; 1: *not at all* - 4: *very much* | - Scores converted to continuous 0-100 scales | Past week |
| **EORTC QLQ-C15-PAL** [22] | - Abbreviated version of the QLQ-C30 for patients in PC | Common symptoms in PC  15 items   - 7 symptoms (e.g., pain, fatigue, weakness) - 2 functional scales (physical / emotional) - 1 Global QoL scale | - 14 items, ordinal scales; 1: *not at all* - 4: *very much* - 1 item on overall health / QoL; 1: *not at all* - 7 *excellent* | - Scores converted to continuous 0-100 scales | Past week |
| **ESAS (Edmonton Symptom Assessment System)** [14] | - Symptom measure for patients with cancer | Common symptoms in cancer  9 items   - Core symptoms (e.g., pain, nausea, tiredness, appetite) - 1 optional to be listed by patients | - NRS; 0; *no symptom* – 10 *worst possible symptom* | - Single item numerical scores | Now |
| **EuroQOL-5D**  [19] | - Generic QOL index | HRQOL for clinical and economic appraisal  5 single items   - Functions, health state; (e.g., mobility, self-care, usual activities) - 1 overall health scale | - EQ-5D: 3 severity levels. 1:*no problems - 3: extreme problems/ unable to* - EQ-5D-5L: severity levels: *no problems - 5: extreme problems/unable* - 1 vertical VAS scale; 0-100; *worst to best imaginable health status* | - Single item scores - Overall score | Today |
| **FAACT (Functional Assessment of Anorexia/Cachexia Treatment) 18-item version** [20] | - General QoL measure + anorexia / cachexia specific measure for use in patients with cancer | General part**^2^** and specific part  45 single items; 27 + 18   - 27 items from FACT-G (see below) - 4 FACT-G scales - 18 items on anorexia-cachexia (e.g., taste, weight, getting full, vomiting) | - 5-point Likert-scale; 0: *not at all – 5: very much* | - Subscale scores - Composite score - Overall anorexia / cachexia score | Past 7 days |
| **FAACT (****Functional Assessment of Anorexia / Cachexia Treatment 12-item version** [29] | - General QoL measure + anorexia / cachexia specific ^1^ for use in patients with cancer | General part**^2^** and specific part  39 single items: 27 + 12   - 27 items from FACT-G (see below) - 4 FACT-G scales - 12 items on anorexia-cachexia (e.g., taste, weight, getting full) | - 5-point Likert-scale; 0: *not at all – 5: very much* | - Subscale scores - Composite score - Overall anorexia / cachexia score | Past 7 days |
| **FACT-An** **(Functional Assessment of Cancer Therapy–Anemia scale)** [27] | - General QoL measure + anaemia specific measure for use in patients with cancer | General part**^2^** and specific part  47 single items: 27 + 20   - 27 items from FACT-G (see below) - 4 FACT-G scales - 20 anaemia related symptoms, (e.g., tired, weak, fatigue, energy) | - 5-point Likert-scale; 0: *not at all – 5: very much* | - Subscale scores - Composite score - Overall anaemia score | Past 7 days |
| **FACIT-F13 (Functional Assessment of Cancer Therapy)** [26] | - Fatigue specific QOL measure for use in patients with cancer | 13-items   - fatigue related symptoms | - 5-point Likert-scale; 0: *not at all – 5: very much* | - Subscale scores - Overall fatigue score | Past 7 days |
| **FACT-G** **(Functional Assessment of Cancer Therapy—General)** [20] | - Generic QOL measure for patients with cancer | 27-items   - 4 scales (social, family, emotional, functional well-being). | - 5-point Likert-scale; 0: *not at all – 5: very much* | - Subscale scores - Overall QOL score | Past 7 days |
| **FACT Head/Neck Symptom Index-2 (FHNSI-2) ^2^** [27] | - Specific instrument for H&N cancer | Generic part**^2^** and specific part  39 single items: 27 + 12   - 27 items from FACT-G (see below) - 4 FACT-G scales - 12 items on H&N related symptoms (e.g., dry mouth, pain, breathing) | - 5-point Likert-scale; 0: *not at all – 5: very much* | - Subscale scores - Composite score - Overall H&N specific score | Past 7 days |
| **LASA (Linear Analogue Self-Assessment or Cancer Linear Analogue Scale)** [17] | - Generic QOL measure for cancer | - 5 items: physical well-being, mood, pain, nausea/and vomiting, appetite | - NRS 0-10; 0; *no symptom* – 10 *worst possible symptom* | - Scores often converted to continuous 0-100 scales | Past 7 days |
| **M.D. Anderson Symptom Inventory (MDASI)**  [15] | - Symptom measure for use in cancer | - 13 symptom items (e.g., pain, fatigue, nausea, sleep disturbance) - 6 interference items (e.g. activity, mood, work, relations) | - NRS 0-10; 0: *not present / did not interfere* – 10: *as bad as you can imagine /* *interfered completely* | - Frequencies of symptoms - Frequencies of interference | The last 24 h |
| **QOL-ACD (Quality of Life Questionnaire for Cancer Patients Treated with Anti-Cancer Drugs)** [24] | - Generic measure for QoL during chemotherapy - Nationally validated - Japanese only | - 21 symptom items - 4 scales (functional, physical, mental psychosocial) - 1 Global QOL scale | - 5-point Likert-scale; 0: *best-good -5: -worst/bad* - QOL scale: 5 Faces | - Scale scores - Separate QOL scale (faces) | Last few days |
| **RSCL (Rotterdam Symptom Checklist)** [23] | - Generic QoL measure | - 39 symptom items; (e.g., appetite, nausea, pain, depression) - 4 scales: Physical, psychological distress, functioning, overall | - 4-point descriptive scales; *not at all – very much* | - Scores converted continuous 0-100 scales - Overall QOL score | Past three days or past week |
| **SF-36** **(MOS short-form 36-survey)** [18] | - Generic QoL measure | - 36 items; (e.g., symptoms, distress, functioning overall health, global QoL - 8 multi-item scales, (e.g., mental, social, general health, physical, role, emotional functioning) | Descriptive scales, different anchors   - 5-point scales: *excellent-poor, not at all/none-very severe/extremely, much better-much worse, all of the time-none of the time, definitely true- definitely false* - 3-point scales: *Yes, limited a lot –No, not limited at all* - Dichotomous items: Y/N | - Scores converted continuous 0-100 scales | Past four weeks, a typical day, generally |
| **Spitzer QOL index** [16] | - Generic QOL measure for cancer and chronic diseases survivors | - 5 items; activity, daily life, health perceptions, social support, behaviour. | - Five 3-point VAS scales: 0-2 | - Total score ranging from 0 to 10 | Past week |

**^1^** As described on the webpages of the measures if applicable, or in the original instrument development paper, ^2^  FACT-G constitutes a part of the condition or diagnosis specific instruments (https://www.facit.org/), all listed as described in the papers, ^3^ Abbreviated version
