# Supplementary material for: Quality of life endpoints in cancer cachexia clinical trials: Systematic review 3 of the cachexia endpoints series
Source: J Cachexia Sarcopenia Muscle. 2024 Mar 29;15(3):794–815. doi: 10.1002/jcsm.13453 (PMC11154790; doi:10.1002/jcsm.13453)
Supplement: Supplementary file 4 — Data S4. Supporting Information. [file JCSM-15-794-s002.docx]

**Supplemental file 3.**

**Proposals - How to improve the use and reporting of QOL endpoints from clinical trials in cancer cachexia.**

| **Issue** | **Frequent limitation** | **Recommendation** |
| --- | --- | --- |
| Selection of measures | - Most studies use multiple measures of QOL | - Select well-validated measures and justify the selection - Avoid ad-hoc tools - Avoid different QOL assessments within the same study |
| Population | - Patient characteristics and stage of disease are not always considered | - Define the definition of cancer cachexia and/or the inclusion criteria that apply to the trial - Discuss the above in relation to the QOL results - QOL endpoints should reflect the intervention - Cancer cachexia has several stages, in which some QOL issues may be more prevalent than other - Consider respondent burden |
| Statistical considerations | - Often inadequate reporting of statistical measures | - If QOL is the primary endpoint, the sample size should be calculated accordingly. - If QOL is not the primary endpoint, state this and consider increasing the sample size accordingly - Always report effect measures - Define both clinical and statistical significance upfront - Provide associations between the QOL results and other endpoints, both primary and secondary - Adjustments for multiple testing should be performed |
| Reporting of results | - Inconsistent reporting of results | - Provide a thorough description of selection of measures, including items, scales and / or global scores that are selected - Do not use single symptom items to denote Global QOL - Define relevant assessment points at which a change in QOL may be plausible, given the interventions - If QOL is measured together with other primary and/or secondary endpoints, present potential limitations in the discussion - Discuss the fact that QOL scores represent group level results and are not applicable to at the individual patient level. Refer to work on MCIDs ^1^ as necessary |

^1^ Minimally Clinically Important Difference
